# Recurrent Fluorescence of Polycyclic Aromatic Hydrocarbon Isomers: A Comparative Study

**DOI:** 10.1021/acsearthspacechem.5c00283

**Published:** 2025-11-25

**Authors:** Arun Subramani, James N. Bull, Henrik Cederquist, Paul Martini, Henning T. Schmidt, Henning Zettergren, Mark H. Stockett

**Affiliations:** † Department of Physics, Stockholm University, Stockholm 114 21, Sweden; ‡ Chemistry, Faculty of Science, 6106 University of East Anglia, Norwich NR4 7TJ, U.K.; § Centre for Photonics and Quantum Science, University of East Anglia, Norwich NR4 7TJ, U.K.

**Keywords:** polycyclic aromatic hydrocarbons, recurrent fluorescence, electrostatic ion-beam storage, ab-initio molecular dynamics, unimolecular dissociation

## Abstract

Time-dependent unimolecular dissociation rates of the C_12_H_8_ isomers acenaphthylene (ACY) and biphenylene (BPY) cations were measured using a cryogenic electrostatic ion beam storage ring. The neutral, cyano-functionalized tracers of ACY, but not of BPY, have been identified in the interstellar molecular cloud TMC-1 by radioastronomy. For both polycyclic aromatic hydrocarbons (PAHs), dissociation is rapidly quenched by recurrent fluorescence (RF). Master equation simulations including RF rate coefficients based on *ab initio* molecular dynamics calculations reproduce the measured dissociation rates. Only marginal differences in the survival probabilities of ACY and BPY in TMC-1 are indicated by these results, with both cations being stable for vibrational energies up to about 7.6 eV, which is 3 eV above the dissociation threshold energy.

## Graphical abstract

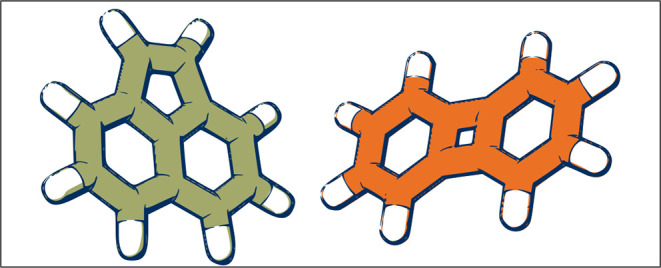

## 1 Introduction

Polycyclic aromatic hydrocarbons (PAHs) have long been thought to be ubiquitous in the interstellar medium (ISM), based on the infrared emission bands observed by astronomers at wavelengths coincident with their characteristic vibrational transition energies.^1^ However, these bands are common to PAHs as a class and have proven to be difficult to assign to specific PAH molecules. It was only in 2021 that the first specific PAHs (cyano-naphthalene, indene, and cyano-indene) were identified in space in the Taurus molecular cloud, TMC-1 by comparing astronomical microwave spectra with known rotational emission lines.^2–5^ More recently, several larger cyano-functionalized PAHs have been identified by the same methods.^6–9^ Among these are two isomers of cyano-acenaphthylene C_12_H_7_CN. The underlying PAH, acenaphthylene (ACY, Figure 1), has similar 5- and 6-membered carbon rings as the previously identified naphthalene^2^ and indene^3–5^ species. The observed abundance of cyano-ACY is comparable to that of cyano-functionalized hydrocarbons comprising 1–7 benzenoid rings.^9^ Intriguingly, the same team who identified cyano-acenaphthylene previously reported a nondetection of cyano-functionalized biphenylene (BPN), which has the same chemical formula as ACY but with a 4-membered ring, in the same data set.^10^ Why is one of these two isomeric PAHs so much more abundant than the other one?

**Figure 1:**
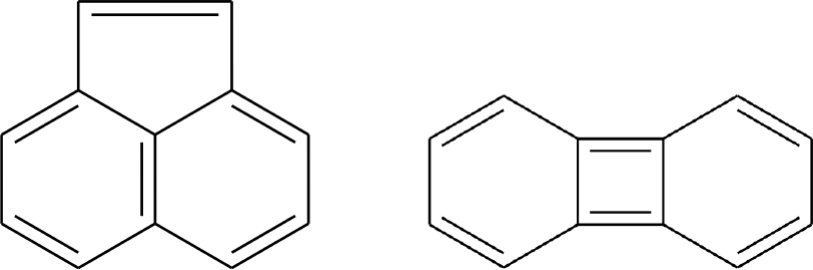
Chemical structures of the C_12_H_8_ isomers acenaphthylene (ACY, left) and biphenylene (BPY, right).

Astrochemical models, which generally reproduce the observed abundances of smaller nitriles in TMC-1 ^11^ underpredict those of PAHs by several orders of magnitude.^2,4^ While conceding that the network of reactions producing cyano-PAHs may be incomplete, the authors of these models also note that the rate of destruction of PAHs may be overestimated. In an alternate version of their model, they find that disabling destructive ion–molecule reactions, the main loss channel in UV-shielded molecular clouds, increased the abundance of cyano-PAHs by 2 orders of magnitude.^2^

It has been argued that one reason astrochemical models may overestimate the destruction rate of PAHs is by the neglect of radiative stabilization^12^ especially by recurrent fluorescence (RF)—the emission of optical photons from thermally excited electronic states.^13,14^ Several recent laboratory studies have found that RF stabilizes many, though not all^15^ PAHs quickly enough to forestall dissociation after ionizing collisions with atomic cations,^12,16,17^ which is believed to be one of the main destruction routes for PAHs in TMC-1.^4,11^

We hypothesize that the difference in the observed abundances of the cyanoACY and cyanoBPN molecules in space may be partially explained by differing RF rates. Here, we test this hypothesis by measuring the time-dependent dissociation rates of the underlying pure hydrocarbon PAHs. While it is the cyano-substituted species that have been identified in TMC-1, this is mainly due to the substituted versions’ much higher dipole moments, which makes them easier to detect with radioastronomy even though their abundances are orders of magnitude lower than pristine PAHs.^5^ The bimolecular rate coefficient for the barrierless, exothermic CN-substitution reaction yielding the cyano-functionalized PAHs is generally assumed to be on the order of 10^–10^ cm^3^s^–1^, with only minor dependence on the size of the underlying PAH.^2,4,8,9,18^ We thus assume that the cyanoPAHs are tracers reflecting the (much higher) abundance of the underlying PAHs, as was demonstrated in the case of cyano-indene in TMC-1.^5^

## 2 Methods

### 2.1 Experiments

Experiments were conducted at the DESIREE (Double ElectroStatic Ion Ring ExpEriment) ion-beam storage ring facility^19,20^ following procedures established in earlier experiments.^12^ ACY and BPN cations were separately formed in an electron cyclotron resonance (ECR, Panteknik Monogan) ion source through a combination of high-energy electron impact ionization and charge exchange with He ions in the ECR plasma. Ensembles of ions with broad distributions of internal energy were extracted from the source, accelerated to 80 keV (316 km/s), mass selected with a bending magnet, and stored using electrostatic deflectors in the storage ring depicted in Figure 2. Neutral fragments emitted from vibrationally hot ions in the lower straight section of the ring (see Figure 2) were counted using a 75 mm diameter Z-stack microchannel plate (MCP) detector (Photonis), backed by a P46 phosphor screen anode.^21^ Light from the phosphor was imaged onto a TimePix3 hybrid pixel detector (Amsterdam Scientific)^22^ which recorded the position of each hit as well as its time of arrival relative to the moment the ions were extracted from the ion source.

**Figure 2:**
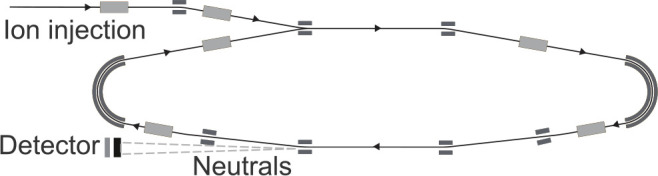
DESIREE electrostatic ion beam storage ring. Ion beams follow the trajectories indicated by the arrows. The blocks represent electrostatic deflectors and focusing elements. The circumference of the stored ions' orbit is 8.6 m. Neutral fragments of ions dissociating in the lower straight section continue with a high velocity to the detector.

### 2.2 Modeling

We model the measured neutral count rate using a well-established master equation approach.^23,24^ The dissociation rate coefficient *k* ^diss^(*E*) is approximated using the so-called inverse Laplace transform formula:^25^

1 $$k^{\mathrm{diss}}\left(E\right)=A^{\mathrm{diss}}\frac{\rho \left(E-E_{a}\right)}{\rho \left(E\right)}$$

where *E* is the total vibrational energy (not including zero-point energy), *A* ^diss^ is a constant, *E*_*a*_ is the activation energy, and ρ­(*E*) is the density of vibrational levels. The level density is computed using the Beyer–Swinehart (BS) algorithm^26^ with vibrational frequencies calculated using Density Functional Theory (DFT). We used B3LYP/N07D DFT methodology^27,28^ that has been shown to accurately predict the vibrational spectra of PAHs.^29,30^ While the BS algorithm assumes harmonically spaced vibrational levels, we use vibrational frequencies computed using the anharmonic VPT2 approximation as implemented in Gaussian16, as this has been shown to more accurately reproduce measured vibrational relaxation rates using the statistical model described below.^31,32^

The infrared (IR) photon emission rate coefficients $k_{s}^{IR}\left(E\right)$ are calculated using the simple harmonic cascade (SHC) approximation:

2 $$\begin{array}{ll} k_{s}^{IR}\left(E\right) & =A_{s}^{IR}\overset{v\leq E/h\nu_{s}}{\underset{v=1}{\sum }}v\frac{\rho_{s}\left(E-h\nu_{s}\right)}{\rho \left(E\right)}\\ & =A_{s}^{IR}\overset{v\leq E/h\nu_{s}}{\underset{v=1}{\sum }}\frac{\rho \left(E-vh\nu_{s}\right)}{\rho \left(E\right)} \end{array}$$

where *v* is the vibrational quantum number, and *h*ν_*s*_ and $A_{s}^{IR}$ are the energy and Einstein coefficient of the *v* = 1 to *v* = 0 transition in vibrational mode *s*, from our DFT calculations. In the first expression, the factor of *v* inside the summation accounts the scaling of the Einstein coefficient with occupation number, and ρ_*s*_(*E*) is the vibrational density of states calculated with mode *s* excluded.^33^ It is numerically equivalent to the second expression^34^ which is faster to calculate. The total vibrational radiative cooling rate $k_{\mathrm{tot}}^{IR}=\underset{s}{\sum }k_{s}^{IR}$ . This model, like the BS algorithm, assumes harmonically spaced vibrational levels and considers only transitions from *v* to *v* – 1.

The RF emission rate coefficients are^14^

3 $$k_{j}^{RF}\left(E\right)=A_{j}^{RF}\frac{\rho \left(E-E_{j}\right)}{\rho \left(E\right)}$$

where *E*_*j*_ and $A_{j}^{RF}$ are the transition energy and Einstein coefficient of the D_*j*_ ←D_0_ electronic transition. The total electronic radiative cooling rate $k_{\mathrm{tot}}^{RF}=\underset{j}{\sum }k_{j}^{RF}$ . Equation 3 tends to severely underestimate measured RF rates for PAHs when used with values of $A_{j}^{RF}$ from standard quantum chemistry calculations performed at equilibrium molecular geometries. Transitions from D_1_ to D_0_ are generally forbidden for PAH radical cations but can still dominate RF rates due to their low activation energy *E*_1_. Computations that include Herzberg–Teller coupling have been shown to yield values of $A_{j}^{RF}$ more in line with experiments, but such calculations are difficult for open-shell species. Recently, a new approach was introduced based on *ab initio* molecular dynamics (AIMD) simulations of molecules with vibrational energies commensurate with those probed in experiments.^15,17^ This approach gives good agreement with measured RF rates without the difficulties of Herzberg–Teller calculations.

AIMD trajectories were performed in ORCA 6.1.0 ^35^ at 9 vibrational energies *E*_*sim*_ ranging from 1.2 to 10.2 eV. At each step (0.5 fs) in the trajectory, the transition energies and oscillator strengths of the lowest three optical transitions are computed at the ωB97X-D/cc-pVTZ level of DFT.^36^ The $k_{j}^{RF}\left(E_{\mathrm{sim}}\right)$ coefficients are calculated at each point and then averaged over the trajectory. Equation 3 is then fit to these discrete points to obtain effective values of *E*_*j*_ and $A_{j}^{RF}$ to be used in the master equation simulation. A scaling factor was applied to the transition energies prior to averaging, such that the effective *E*_*j*_ values from the fit are brought into alignment with measured values. The need for energy scaling is the main weak point of the AIMD method, given that it is too computationally expensive to deploy better methods, e.g., EOM-CCSD, for each step in a trajectory.

In the simulation, the initial distribution of vibrational energies *g*(*E*,*t* = 0) is assumed to follow a Boltzmann distribution corresponding to some temperature *T*. The energy distribution of the ensemble evolves according to the master equation:

4 $$\begin{array}{l} \frac{d}{dt}g\left(E,t\right)=-k^{\mathrm{diss}}\left(E\right)g\left(E,t\right)\\ +\underset{s}{\sum }\left[k_{s}^{IR}\left(E+h\nu_{s}\right)g\left(E+h\nu_{s},t\right)-k_{s}^{IR}\left(E\right)g\left(E,t\right)\right]\\ +\underset{j}{\sum }\left[k_{j}^{RF}\left(E+E_{j}\right)g\left(E+h\nu_{j},t\right)-k_{j}^{RF}\left(E\right)g\left(E,t\right)\right] \end{array}$$

The first term on the right-hand side represents destruction of the ions by dissociation, and the second and third lines represent redistribution of population by vibrational and electronic cooling, respectively. The dissociation rate, which is proportional to the measured neutral product yield, is given by ∫*k* ^diss^(*E*)*g*(*E*,*t*)*dE*.

## 3 Results and Discussion

Figure 3 presents the perpendicular velocity distribution of neutral particles reaching the DESIREE imaging detector. These distributions are obtained by performing an inverse Abel transform on the azimuthally integrated radial intensity distribution in the detector plane.^15^ All neutral events detected within 10 ms after the ions left the ECR source (about 360 rpm in the storage ring) are included in the distributions. For both ACY^+^ and BPY^+^, the distribution extends beyond the limit set by the edge of the MCP detector. This is consistent with the mass spectroscopic study of West et al.^37^ who found that H atom loss was the dominant primary dissociation channel of ACY^+^, with C_2_H_2_-loss making a minor contribution. Given the low lab-frame velocity of the heavy PAH^+^ reactant stored in the ring, the light H product has time to travel beyond the detector, even when only small amounts of kinetic energy are released in the reaction.

**Figure 3:**
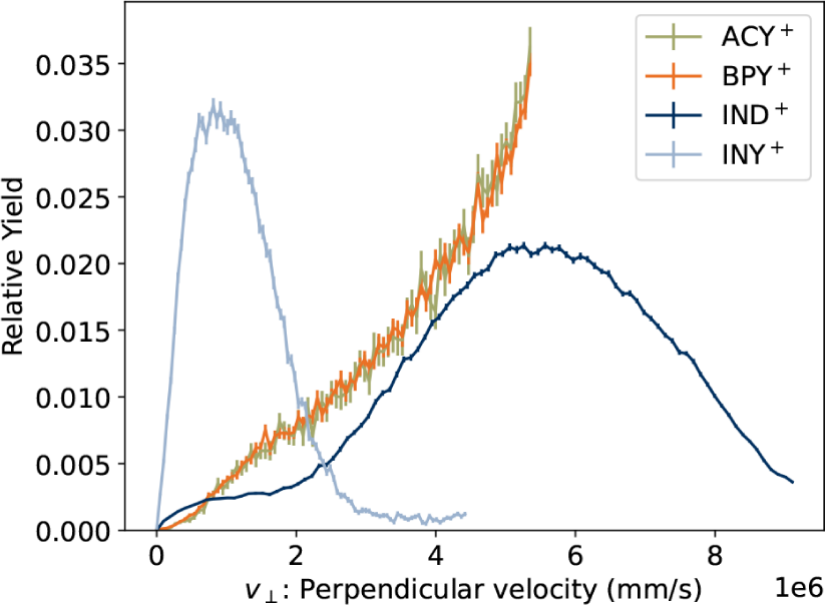
Neutral product velocity distributions for ACY^+^ and BPY^+^. The distribution is cut off at the edge of the detector. For comparison, the distributions previously measured for indene^15^ (IND^+^, $C_{9}H_{7}^{+}$ ), which decays primarily though H-loss, and indenyl^17^ (INY^+^, $C_{9}H_{7}^{+}$ ), which decays primarily through C_2_H_2_-loss.

For comparison, the velocity distribution for indene cations (IND^+^, $C_{9}H_{8}^{+}$ ), measured previously at DESIREE^15^ is also given in Figure 3. IND^+^ also dissociates primarily via H-loss^38^ but, because IND^+^ is lighter than the present cations, the products mostly remain within the range detectable in DESIREE. Also included for comparison is the velocity distribution for C_2_H_2_-loss from indenyl cations (INY^+^, $C_{9}H_{7}^{+}$ ).^17^ The kinetic energy of the fragments is on the order of a few hundred meV in both cases. While a quantitative analysis of the kinetic energy release distribution is clearly not possible in the present experiment, it is clear that both ACY^+^ and BPY^+^ dissociate mainly by H-loss, in agreement with the measurements of West et al.^37^ for ACY^+^.

The time-dependent dissociation rates *R*(*t*) of ACY and BPY cations are plotted in Figure 4. A constant background of about 4 s^–1^ due to detector dark noise has been subtracted. The measured dissociation rate is proportional to the dissociation rate coefficient *k*_diss_(*E*), averaged over the vibrational energy distribution *g*(*E*,*t*) of the ensemble:

5 R(t)∝∫k(E)g(E,t)dE

**Figure 4:**
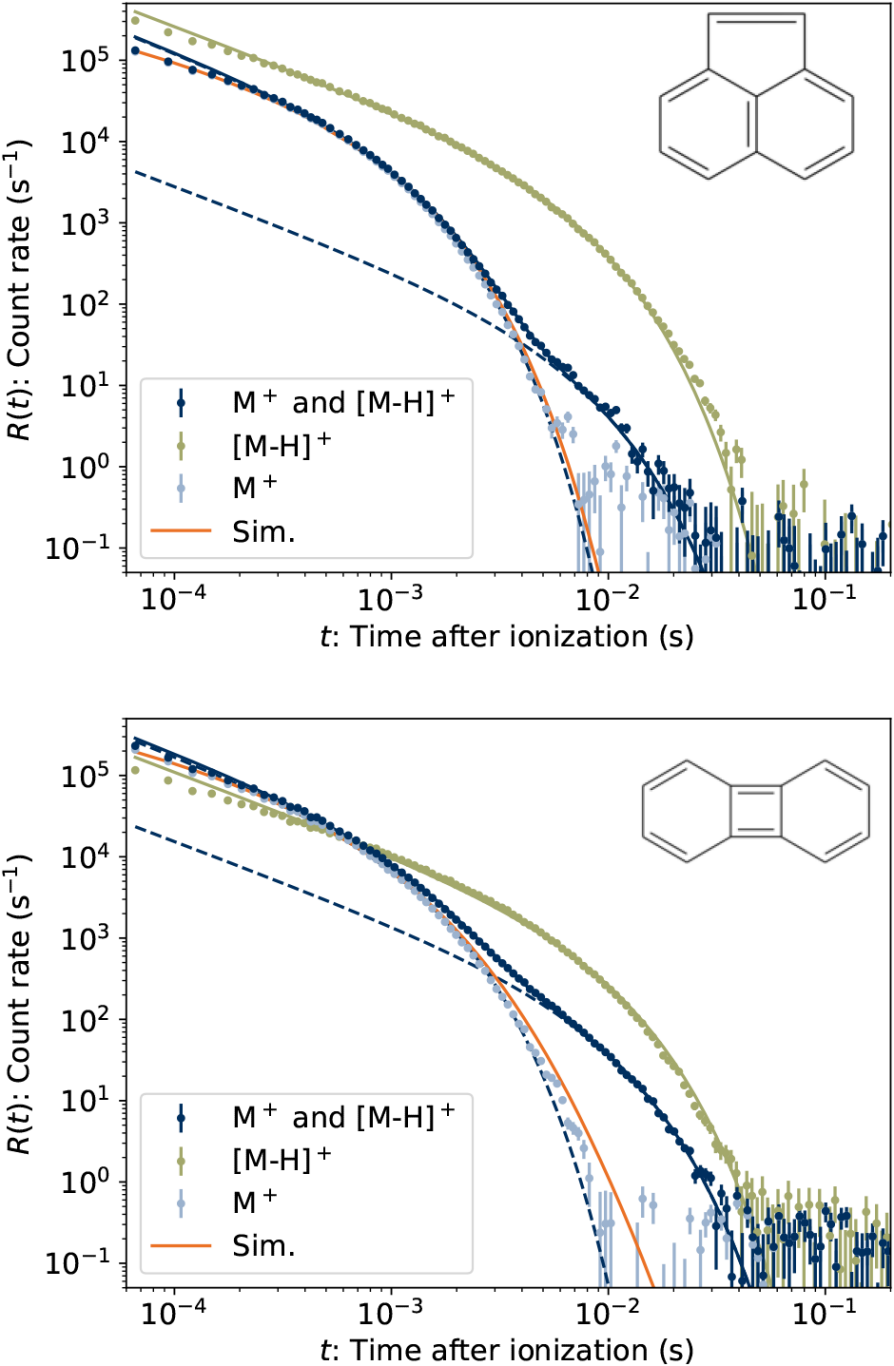
Neutral product count rates *R*(*t*) for stored beams of M^+^=ACY^+^ (upper) and BPY^+^ (lower). The raw data include contributions from the reactant M^+^ and the product [M-H]^+^ containing one ^13^C atom, both with *m*/*z* = 152. The *R*(*t*) values for [M-H]^+^ (with no ^13^C) were measured separately by selecting *m*/*z* = 151. The dashed curves show that the two components (eq 6) fit to the raw data. The data labeled M^+^ are the raw data with the fitted contribution of [M-H]^+^ subtracted. The curves labeled “Sim” are the best-fitting results of our master equation simulation.

In the limit of a flat energy distribution, subject to radiative cooling, eq 5 can be approximated as

6 $$R\left(t\right)\propto t^{-1}e^{-k_{c}t}$$

where *k*_*c*_ is the critical rate coefficient where dissociation competes with radiative cooling.^39^ The measured rates for ACY and BPN both present more complex two-component character. Similar behavior has been observed previously in experiments where different destruction channels are accessible to subpopulations of the stored ion ensemble with different angular momenta.^40,41^ Here, we attribute the second component to contamination of the reactant beam ^12^C_12_ $H_{8}^{+}$ with its isotopologue bearing a single ^13^C, having already lost an atomic H fragment prior to mass selection, i.e., ^13^C^12^ $C_{11}H_{7}^{+}$ . Both species are transmitted by the bending magnet set to the reactant *m*/*z* = 152, giving rise to the two-component character of the measured *R*(*t*), labeled “M^+^ and [M-H]^+^” in Figure 4. Similar effects have been observed previously in experiments with PAH cations in electrostatic storage devices^15,24^ but not with such cleanly separable components.

We account for the contamination through a separate measurement in which the ^12^C_12_ $H_{7}^{+}$ product ion is instead mass-selected (*m*/*z* = 151) and stored. These results are plotted in the respective panels of Figure 4 as “[M-H]^+^,” and indeed closely resemble the second, more slowly quenched component of the *m*/*z* = 152 result. To the [M-H]^+^ data, we fit eq 6. Holding the fitted *k*_*c*_ parameter of the slow component fixed, a two-component fit was made to the “M^+^ and [M-H]^+^” data. The results are listed in Table 1. The first 200 μs have been excluded from all fits to minimize the impact of detector saturation on the *k*_*c*_ estimates.

**Table 1:** Model *k*_*c*_ Parameter Estimates (s^–1^, Uncertainty in Last Digits in Parentheses) from Least-Squares Fits of eq 6 to the Measured Dissociation Rates *R*(*t*) of ACY and BPY Cations, See Figure 4

M	M^+^	[M-H]^+^
ACY	1230(10)	194(1)
BPY	1050(10)	146(2)

In both cases, the slow component, attributed to the isotopologous [M-H]^+^ contamination, is characterized by a critical rate coefficient on the order of 100 s^–1^, which is typical of vibrational radiative cooling of PAH cations,^15,32,42^ and anions.^43^ The faster component, due to the reactant ion M^+^, have much higher values of *k*_*c*_, among the highest reported for PAH cations to date.^44,45^ Such rapid stabilization can be affected only by recurrent fluorescence. The inferred dissociation rate of M^+^, found by subtracting the fitted slow component from the “M^+^ and [M-H]^+^” data, is plotted in Figure 4.

While the measured *R*(*t*) for the two reactant ions is qualitatively similar, the fitted values of *k*_*c*_ are significantly different. This is in contrast to a previous study of the $C_{10}H_{8}^{+}$ isomers naphthalene and azulene, where the *k*_*c*_ values were within mutual error bars.^16^ In that case, the barrier to isomerization between the isomers lies below the dissociation threshold energy, giving rise to a pre-equilibrium mixture of isomers in the stored beam and indistinguishable dissociation rates. While a full mapping of the potential energy surface connecting the ACY^+^ and BPY^+^ is beyond the scope of this work (see Banhatti et al. for some discussion,^46^ from the present results it does not appear that isomerization between them is fast compared to dissociation.

Effective transition energies and oscillator strengths for ACY and BPY cations derived from our AIMD simulations (Section 2.2) are given in Tables 2 and 3, respectively. As described in Section 2.2, scaling factors are needed to bring the AIMD transition energies, which are calculated at the ωB97X-D/cc-pVTZ level of DFT, into alignment with experimental values. Unfortunately, no electronic absorption spectra of the gas-phase ions are available in the literature. Values taken from matrix isolation spectroscopy (MIS)^47^ performed on the cations and photoelectron spectroscopy of the neutral species^48,49^ (where the spacing between measured ionization energies gives an approximation of the cation’s electronic transition energies) are included in the tables, as are the results of earlier TD-DFT calculations using the BLYP functional and 6–31G** basis set.^50^ We performed EOM-CCSD/cc-pVTZ^51^ calculations of the vertical transition energies at the cationic ground-state geometry, which reproduce some of the measured band maxima. A rather high scaling factor of 1.26 was needed to bring the energy of the D_1_ ←D_0_ transition in ACY^+^ into agreement with the experiment. A smaller factor of 0.9 was used for BPY^+^.

**Table 2:** Vertical Electronic Transition Energies in eV (Oscillator Strengths in Parentheses) of Acenaphthylene Radical Cation ACY^+^ from Photoelectron Spectroscopy (PES)^48^ Matrix Isolation Spectroscopy (MIS)^47^ Time-Dependent Desnity Functional Theory (TD-DFT)^50^ Equation of Motion Coupled Cluster Singles and Doubles Calculations (EOM-CCDS, Present Work) and *Ab-Initio* Molecular Dynamics (AIMD, Present Work) Simulations^a^

	PES^48^	MIS^47^	TD-DFT^50^	EOM-CCSD	AIMD
D_1_	0.77	0.80	0.63(0.001)	1.04	0.79(0.003)
D_2_		1.15		1.49	1.49(0.002)
D_3_	2.65	2.53	2.55(0.001)	3.02	2.51(0.008)

a The AIMD values are fits to averages over multiple AIMD trajectories, with the transition energies having been scaled by a factor of 1.26.

**Table 3:** Vertical Electronic Transition Energies in eV (Oscillator Strengths in Parentheses) of Biphenylene Radical Cation BPY^+^ from Photoelectron Spectroscopy (PES)^48,49^ matrix Isolation Spectroscopy (MIS)^47^ Time-Dependent Desnity Functional Theory (TD-DFT)^50^ Equation of Motion Coupled Cluster Singles and Doubles Calculations (EOM-CCDS, Present Work) and *Ab-Initio* Molecular Dynamics (AIMD, Present Work) Simulations^a^

	PES^48,49^	MIS^47^	TD-DFT^50^	EOM-CCSD	AIMD
D_1_	1.29	1.35	1.16(0.001)	1.98	1.50(0.031)
D_2_	2.07		1.80(0.000)	2.46	1.87(0.041)
D_3_	2.47	2.33	2.47(0.092)	2.77	2.23(0.065)

a The AIMD values are fits to averages over multiple AIMD trajectories, with the transition energies having been scaled by a factor of 0.9.

In the case of ACY^+^, the effective oscillator strengths resulting from our AIMD simulations are significantly, but not dramatically, higher than in the earlier TD-DFT results. The enhancement of the D_1_ →D_0_ and D_2_ →D_0_ fluorescing transitions of BPY^+^ is much more pronounced, suggesting a vibronic coupling stronger than that for ACY^+^.

Figure 5 presents our calculated rate coefficients for RF and IR radiative cooling for ACY^+^ and BPY^+^. The RF rate coefficients (eq 3) are computed using the transition energies and oscillator strengths from our AIMD simulations (Tables 2 and 3). For both ions, the RF rate coefficient *k*_RF_ is dominated by the D_1_ →D_0_ fluorescing transition.

**Figure 5:**
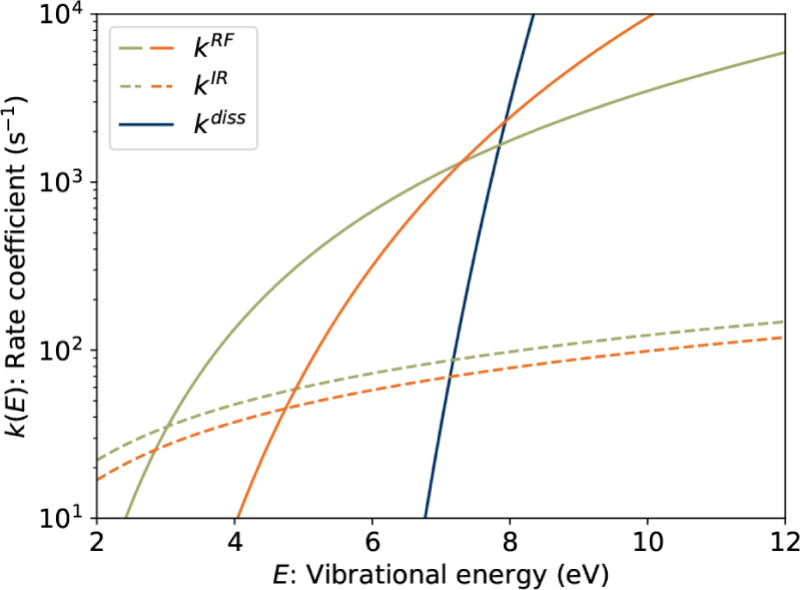
Modeled rate coefficients for RF (solid) and IR (dashed) radiative cooling for ACY^+^ (green) and BPY^+^ (orange). The dissociation rate coefficient (solid blue) is adapted from West et al.^52^ and is assumed to be the same for both ions.

The dissociation rate coefficient for H-loss from ACY^+^ was previously reported by West et al.^52^ who used variational transition state theory to fit measured breakdown diagrams. For consistency, we have refitted their tabulated rate coefficients with our simpler model represented by eq 1. We find that eq 1 with *E*_*a*_ = 4.68 eV and *A* ^*diss*^ = 6.1× 10^15^ s^–1^ reproduces the rate coefficient of West et al. across the energy range of interest. Our refit model of *k* ^diss^(*E*) is plotted in Figure 5. As the dissociation energy for H-loss is found to not vary significantly with PAH size or structure^52,53^ we assume *k* ^*diss*^(*E*) to be the same for both ACY^+^ and BPY^+^. It should be noted that the time-of-flight mass spectrometry technique used by West et al. is sensitive only to dissociation reactions occurring on the microsecond time scale,^52^ i.e., to ions with much higher internal energies than those probed in the present storage ring experiment. Previous studies of H-loss from PAH cations have found reasonable agreement between the two different methods.^15,24,38,52^

We performed master equation simulations (eq 4) using the rate coefficients plotted in Figure 5 for both ACY^+^ and BPY^+^. We assumed the initial energy distribution *g*(*E*,*t* = 0) followed a Boltzmann distribution characterized by temperature *T*. We varied *T* to obtain the best fit of the simulated neutral yield to the measured *R*(*t*) with an arbitrary scaling factor accounting for the unknown detection efficiency. These best fitting simulations, with *T* = 2060(70) K for ACY^+^ and *T* = 1980(70) K for BPY^+^, are plotted in Figure 4. Such temperatures are typical for PAH cations produced in ECR ion sources.^24^

The critical energy *E*_*c*_ where *k* ^RF^(*E*_*c*_)≈*k* ^diss^(*E*_*c*_) is about 7.6 eV for both ions. The RF rate coefficients at this energy are on the order of 1 × 10^3^ s^–1^, similar to the fitted values of *k*_*c*_ in Table 1. While ACY^+^ has a lower activation energy for RF (the D_1_ → D_0_ transition energy), the D_1_ → D_0_ transition probability for BPY^+^ is an order of magnitude higher, resulting in coincidentally similar RF rate coefficients near the critical energy. The IR emission rate coefficients are an order of magnitude lower. For both ions, RF is the dominant radiative stabilization mechanism and the leading unimolecular process across a broad range of internal energies.

## 4 Astrochemical Implications

ACY^+^ and BPY^+^, despite having rather different chemical and electronic structures, have similar dissociation and radiative stabilization rates. According to our kinetic modeling, both ions will be effectively radiatively stabilized if their internal energies remain below the critical energy of 7.6 eV. In TMC-1, neutral PAHs are supposed to be destroyed following ionizing collisions with atomic and molecular cations, including H^+^, C^+^, and He^+^ at 10 K thermal velocities.^2,4^ The molecular cations serve as proton donors^4,54^ these reactions are not considered here. For the atomic cations, radical PAH cations are formed with internal energies with a maximum value given by the difference in the ionization potentials:

7 $$\mathrm{PAH}+B^{+}\to {\mathrm{PAH}}^{+*}+B$$

where B is H, C, or He. Given the ionization potentials of ACY and BPY (8.1 and 7.6 eV, respectively),^37,48,49^ the charge transfer reaction eq 7 with H^+^ or C^+^ never deposits sufficient energy to induce dissociation of the PAH^+^ product. While large energy deposits in ionizing collisions with He^+^ are energetically possible, charge transfer in this case would likely occur at quite large distances, depending on the positions of the crossings of the potential energy curves, resulting in minimal vibrational excitation of the PAH^+^ product. As in previous studies of PAH cation radiative stabilization, we find that recurrent fluorescence halts the destruction of PAHs in molecular clouds following electron transfer at the PAH radical cation. Further reactions involving PAH cations, such as electron–ion recombination and cation–anion mutual neutralization^55–57^ should be included in astrochemical models to ascertain whether “recycling” of PAH cations back into the neutral PAHs identified by radio astronomy can contribute to the neutrals’ observed abundances.

While not considered to be a dominant process in TMC-1 ^2,4^ photodissociation of PAH cations induced by ultraviolet radiation is important in more diffuse interstellar media.^42^ In these regions, RF may play a role in determining the lower size limit for PAH stability. The critical energy *E*_*c*_ for radiative stabilization of fully *sp* ^3^-hybridized PAH cations ranges from about 5.8 eV for indenyl^17^ and naphthalene^16^ through 7.6 eV for the present targets, to about 10.6 eV for tetracene^45^ and perylene.^24^ The last value is greater than the Lyman-α photon energy, which dominates many astronomical radiation fields, although we note that the critical energy corresponds to a survival probability of 50%. Astrochemical modeling is required to predict the lifetimes of PAH cations in various environments.

Our hypothesis was that the detection of cyano-ACY and the nondetection of cyano-BPY in TMC-1 could be rationalized by different radiative stabilization rates of the underlying ACY^+^ and BPY^+^. Having measured the rates, we found that this is not the case. Dissociation following charge transfer with H^+^ and C^+^ is energetically closed for both ions, and the marginal difference in the survival probability following He^+^ collisions seems unlikely to be significant.

We suggest two additional hypotheses to explain the differing abundances of cyano-ACY and cyano-BPY. First, the mechanisms and rates of formation of the underlying PAHs may be very different. For example, ACY^+^ has been shown to be a common product of dissociative ionization of larger PAHs.^46^ Thus, ACY, which is also the lowest-energy C_12_H_8_ isomer, may be more likely to be formed in a top-down formation scenario.

Second, the radiative stabilization rates of the cyano-functionalized cations may be significantly different from those of the underlying PAHs. By lowering the symmetry of the molecule, substitution increases the transition probability for the lowest-energy electronic transition, leading to faster cooling. On the other hand, the threshold dissociation energy is lowered, as the CN group is more weakly bound than a hydrogen atom would be. This latter effect is less important for larger PAHs, which have many more H atoms to lose.^58^ The balance of these effects is not trivial to predict. The critical rate coefficient for radiative stabilization of cyano-indene is higher than that for indene^15^ while the opposite is true for cyano-naphthalene and naphthalene.^12,16^ Thus, functionalization could possibly increase the difference in stability and contribute to the differing observed abundances.

## 5 Conclusions

Recurrent fluorescence is a fundamental unimolecular process and has been found to be the dominant radiative stabilization mechanism of nearly every PAH cation, which has been subject to experimental study. RF rate coefficients are not trivial to predict using standard quantum chemistry approaches due to the importance of vibronic coupling effects. In the present case, a prediction based on basic TD-DFT calculations would indicate much more rapid radiative stabilization of ACY^+^ than of BPY^+^. Our AIMD approach, validated by comparison to the present experiments, finds more similar stabilization rates owing to stronger vibronic coupling in BPY^+^.

While the marginal difference in stabilization rates cannot explain the difference in observed abundances of cyano-ACY and cyano-BPY in TMC-1, we find that both underlying PAHs are resilient against dissociation following charge exchange with atomic cations. Improved understanding of the next steps in the chemical evolution of these species, including laboratory measurements of neutralization reactions, is needed to improve astrochemical models.
